# Creation of a watermelon haploid inducer line via *ClDMP3*-mediated single fertilization of the central cell

**DOI:** 10.1093/hr/uhad081

**Published:** 2023-04-19

**Authors:** Xiner Chen, Yuxiu Li, Gongli Ai, Jinfan Chen, Dalong Guo, Zhonghou Zhu, Xuejie Zhu, Shujuan Tian, Jiafa Wang, Man Liu, Li Yuan

**Affiliations:** State Key Laboratory of Crop Stress Biology for Arid Areas, College of Horticulture, Northwest A&F University, Yangling, 712100, Shanxi, China; State Key Laboratory of Crop Stress Biology for Arid Areas, College of Horticulture, Northwest A&F University, Yangling, 712100, Shanxi, China; State Key Laboratory of Crop Stress Biology for Arid Areas, College of Horticulture, Northwest A&F University, Yangling, 712100, Shanxi, China; State Key Laboratory of Crop Stress Biology for Arid Areas, College of Horticulture, Northwest A&F University, Yangling, 712100, Shanxi, China; College of Horticulture and Plant Protection Henan University of Science and Technology, 471000, Luoyang, China; Luoyang Nongfa Agricultural Technology Co., LTD, 471100, Luoyang, China; Luoyang Nongfa Agricultural Technology Co., LTD, 471100, Luoyang, China; State Key Laboratory of Crop Stress Biology for Arid Areas, College of Horticulture, Northwest A&F University, Yangling, 712100, Shanxi, China; Shenzhen Research Institute, Northwest A&F University, Shenzhen, 518000, Guangdong, China; State Key Laboratory of Crop Stress Biology for Arid Areas, College of Horticulture, Northwest A&F University, Yangling, 712100, Shanxi, China; Shenzhen Research Institute, Northwest A&F University, Shenzhen, 518000, Guangdong, China; State Key Laboratory of Crop Stress Biology for Arid Areas, College of Horticulture, Northwest A&F University, Yangling, 712100, Shanxi, China; Shenzhen Research Institute, Northwest A&F University, Shenzhen, 518000, Guangdong, China; State Key Laboratory of Crop Stress Biology for Arid Areas, College of Horticulture, Northwest A&F University, Yangling, 712100, Shanxi, China; Shenzhen Research Institute, Northwest A&F University, Shenzhen, 518000, Guangdong, China

## Abstract

The use of doubled haploids is one of the most efficient breeding methods in modern agriculture. Irradiation of pollen grains has been shown to induce haploids in cucurbit crops, possibly because it causes preferential fertilization of the central cell over the egg cell. Disruption of the *DMP* gene is known to induce single fertilization of the central cell, which can lead to the formation of haploids. In the present study, a detailed method of creating a watermelon haploid inducer line via *ClDMP3* mutation is described. The *cldmp3* mutant induced haploids in multiple watermelon genotypes at rates of up to 1.12%. These haploids were confirmed via fluorescent markers, flow cytometry, molecular markers, and immuno-staining. The haploid inducer created by this method has the potential to greatly advance watermelon breeding in the future.

## Introduction

Generating haploid plants to obtain pure doubled haploid (DH) lines is one of the most efficient breeding strategies in modern agriculture. The simultaneous genetic fixation of every locus within a single generation enables breeders to avoid the time-consuming conventional requirement for extensive selfing or backcrossing before homozygous breeding-ready lines can be obtained. Currently, haploids can be obtained through either *in vitro* or *in vivo* methods. *In vitro* methods are mainly based on culturing haploid gametophytic tissues or organs, including anthers, microspores or ovaries, to obtain haploid plants. However, *in vitro* haploid methods are often affected by genotype, developmental stage, culture condition, physiological status and stress pretreatment of the plant material. *In vivo* induction generates haploids through interspecific or intraspecific crosses but results in a low frequency of haploid induction. Parthenogenesis, on the other hand, has naturally occurred in over 400 plant species and is characterized by the development of egg cells into haploid embryos without fertilization [1]. Additionally, haploids can sometimes be induced after pollination with pollen treated with radiation, chemicals or high temperatures [2].

Haploid inducer lines are increasingly used to simplify haploid induction process and new techniques are constantly being developed to generate such lines as the understanding of reproductive biology increases. Generally, haploid inducer lines induce parental genome elimination during the first division of zygotic embryos or parthenogenesis of egg cells during fertilization [2]. For example, the maize inbred line STOCK6 has been reported to induce haploids at rates of 2.3–3.2% [3]. Map-based cloning revealed that a 4-bp insertion in a phospholipase gene, *MATRILINEAL*/*PHOSPHOLIPASE A1*/*NOT LIKE DAD* (*MTL*/*PLA1*/*NLD*), was responsible for the haploid induction ability of STOCK6 [4–6]. MTL protein was shown to localize to the sperm-cell membrane, indicating that it is likely involved in the interaction of sperm cell and egg cell during fertilization. Mutation of *Zea mays PHOSPHOLIPASE D3* (*ZmPLD3*), which encodes a member of the phospholipase D subfamily, was also shown to trigger maternal haploids during fertilization in maize. Furthermore, the *zmpld3* mutant was found to synergistically increase the efficiency of haploid induction mediated by the *zmpla1* mutant [7]. Additionally, manipulation of the centromeric histone protein CenH3 can cause haploid induction through parental genome elimination, possibly because modified CenH3 protein results in slower chromosome movement during the first division of zygotic embryos, leading to the elimination of one parental genome [8]. Point mutations in the histone folding domain or centromere-targeting domain of CenH3 protein were also shown to induce haploids in *Arabidopsis* [9–11]. The CenH3-mediated haploid induction system has been successfully applied to many different monocot crops, including maize, wheat and others [12–14]. BABY BOOM (BBM), an AP2 transcription factor family protein, can initiate maternal parthenogenic embryogenesis without fertilization and leads to haploid formation in rice, maize, millet and *Arabidopsis* [15–18]. By combining a *Meiosis instead of Mitosis* (*MiMe*) system with *BBM*, researchers have shown that hybrid vigor can be efficiently fixed across generations through synthetic apomixis in rice [19, 20]. However, ectopic expression of the *BBM* gene in egg cells is a prerequisite, so *BBM-*mediated parthenogenic haploids are transgenic and have limited application in plant breeding.

Watermelon (*Citrullus lanatus*) is an important fruit crop worldwide that is consumed for its nutritional value and flavor [21]. Long-term artificial selection of watermelon had led to genetic narrowing, and a haploid induction system is urgently needed to advance traditional breeding by enabling the creation of valuable pure DH lines [22, 23]. Irradiated pollen grains have been demonstrated to induce haploid plants in cucurbit crops with relatively low efficiencies [24–26]. This may be because irradiation causes sperm cells to lose the ability to fertilize the egg cell, while maintaining the ability to fuse with the central cell to give rise to the endosperm. In rare cases, unfertilized egg cells could then further develop into parthenogenic haploid embryos. Therefore, we hypothesized that the preferential fertilization of the central cell over the egg cell could possibly induce haploid formation in watermelon.

**Figure 1:**
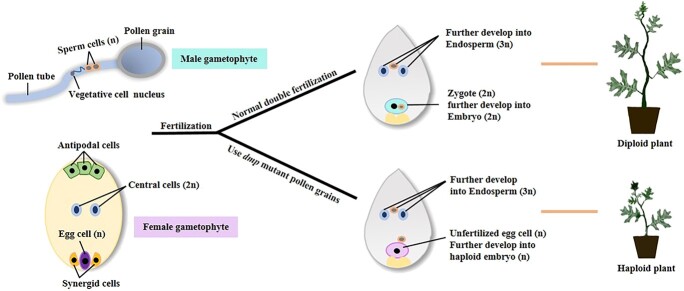
Schematic illustration of the hypothesis of *DMP* mediated haploid induction

To date, several genes have been reported to be involved in sperm-egg fusion, and their mutations can result in single fertilization of the central cell. The *LlDMP9* gene, which encodes a DOMAIN OF UNKNOWN FUNCTION 679 (DUF679) protein localized at the sperm membrane in lily (*Lilium longiflorum*), is involved in the fusion of gametes. Earlier work found that knocking down the *Arabidopsis DMP9* gene resulted in 17.2 ± 1.8% single central cell fertilization events [27]. *DMP8* is another *DUF679* gene that is specifically expressed in pollen, and the *Arabidopsis dmp8/9* double mutant led to single fertilization of the central cell at a rate of 19.04 ± 5.68% [28, 29]. Consistent with our hypothesis that single fertilization of the central cell induces haploids in plant species, the *dmp8/9* double mutant was found to induce haploids in *Arabidopsis* [30]. The maize *zmdmp* mutant was initially identified as an enhancer of the haploid inducer *zmpla1*, which typically induces haploids at low efficiency [31]. The *DMP*-based haploid induction system has been successfully applied to several plant species, including tomato, tobacco, potato, *Brassica napus*, and *Medicago truncatula*, which prompted us to explore the possibility of inducing haploids in watermelon with this same approach [32–36] (Fig. 1).

In the present study, we describe a method for creating a haploid inducer line in watermelon by employing mutations in the *ClDMP3* gene. Mutation of *ClDMP3* caused single fertilization of the central cell over the egg cell, and the *cldmp3* mutant could induce haploids in several different watermelon genotypes, with rates ranging from 0.51% to 1.12%. The generated haploids were confirmed via fluorescent markers, flow cytometry, molecular markers and immuno-staining. These findings have the potential to significantly accelerate future watermelon breeding efforts.

## Results

### Identification of the *ClDMP3* gene in watermelon

**Figure 2:**
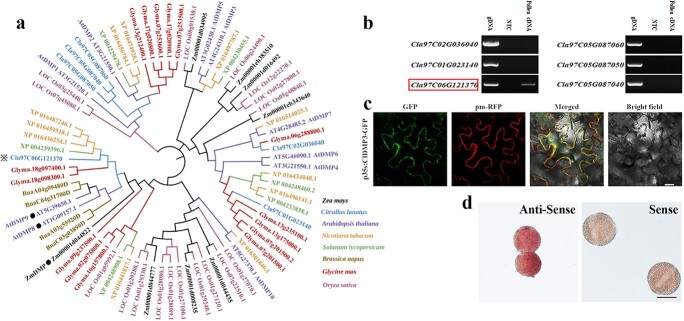
Identification of the *ClDMP3* gene in watermelon. **a.** Phylogenetic analysis of DMP homologs in eight green plant species. ClDMP3 proteins are highlighted with asterisks, and the DMPs in *Arabidopsis* and *Zea mays* are noted with dots. **b.** Expression of watermelon *DUF679* in mature pollen. Genomic DNA (gDNA) and non-template control (NTC) were used as controls for RT-PCR. The *ClDMP3* gene is noted in the red box. **c.** Subcellular localization of ClDMP3-GFP proteins in tobacco leaf. pm-RFP is a plasma membrane marker. **d.** Whole-mount RNA *in situ* hybridization analysis of *ClDMP3* in mature watermelon pollen. Scale bars: 20 μm (c), 50 μm (d).

Six putative DUF679 proteins were identified in the watermelon genome, among which the protein encoded by *Cla97C06G121370* exhibited the highest homology to *Arabidopsis* AtDMP8/9 and maize ZmDMP proteins (Fig. 2a, Table S2). Subsequent expression analysis revealed that *ClDMP3* was the only *DUF679* gene that was specifically expressed in pollen (Fig. 2b, Fig. S1), which was consistent with our pollen RNA-seq data (unpublished data). Subcellular localization analysis indicated that ClDMP3 was localized to the cell membrane (Fig. 2c), which is consistent with a possible role in sperm-egg cell membrane fusion. A whole-mount RNA *in situ* experiment further confirmed the presence of *ClDMP3* transcripts in mature pollen (Fig. 2d). Therefore, *ClDMP3* was chosen for mutagenesis mediated by CRISPR/Cas9 gene editing technology.

### Generation of *cldmp3* mutants

**Figure 3:**
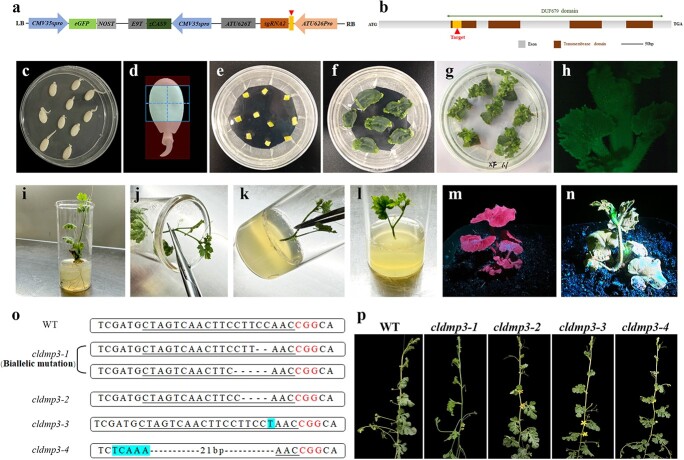
Generation of the *cldmp3* mutants through CRISPR/Cas9 gene editing technology. **a.** The *CRISPR/Cas9* editing vector for *ClDMP3* with a single guide RNA. The red triangle indicates the *ClDMP3* target. **b.** Schematic diagram of the *ClDMP3* gene. **c-n**. Watermelon transformation protocol: (c) Germination of watermelon seeds; (d) Schematic diagram of the cotyledon explant cutting method. The red area represents the part not suitable for severing as explants, which should be removed and discarded. The blue area represents the cutting area; (e) Explants cultivated on the recovery medium; (f) Explants cultivated on the shoot generation medium; (g) New shoots formed after one month of cultivation on the shoot generation medium; (h) Positive transgenic shoots with GFP marker expression; (i) Positive transgenic seedling on the rooting medium; (j-l) Asexual reproduction of positive shoots; (m) Control watermelon seedling without GFP fluorescence; (n) Transgenic positive seedling with green fluorescence. **o.** Target sequence and the actual editing results in different *cldmp* mutant plants. The PAM sequence is shown in red font and the 20 bp target sequence is underlined. Insertions are highlighted in blue. Deletions are shown with black dashed lines. **p.** Comparison of WT and *cldmp3* mutant plants.

A single target was designed against the *ClDMP* coding region and cloned into the CRISPR/Cas9 gene editing vector pBSE402, which contained a 35S-driven Cas9, an AtU6–26 driven SgRNA cassette and a GFP fluorescent marker driven by the 35S promoter (Fig. 3a and b). Stable watermelon transformation was carried out using *Agrobacterium tumefaciens* strain EHA105, and watermelon cotyledon sections were utilized as explants. Additionally, 6-benzyladenine (6-BA) and Timentin were supplied in the medium during the shoot elongation process, and Timentin alone was added to the medium to inhibit the growth of bacteria during the rooting process. Healthy transgenic plants were transferred to soil for further analysis (Fig. 3c-n).

After obtaining the potential transgenic plants, primers were designed to amplify the region spanning the target site (Table S3), and Sanger sequencing was used to further confirm the editing events. Subsequently, Hi-TOM deep sequencing was utilized to reveal the editing types and ratios [37]. Out of 11 positive transgenic plants, 4 plants were identified with valid edits that were either biallelic or homozygous mutations. *cldmp3–1* contained −2/−5 biallelic mutations in the target, while *cldmp3–2* was homozygous for a 4-bp deletion, and *cldmp3–3* was homozygous for a 1-bp insertion at the target site. Additionally, *cldmp3–4* contained a homozygous 21-bp deletion and a 5-bp insertion inside the target region (Fig. 3o). All mutations caused pre-termination of the ClDMP3 protein. Phenotypic analysis did not reveal any obvious abnormalities in the mutant plants during vegetative growth (Fig. 3p).

### Phenotypic analysis of the *cldmp3* mutants

**Figure 4:**
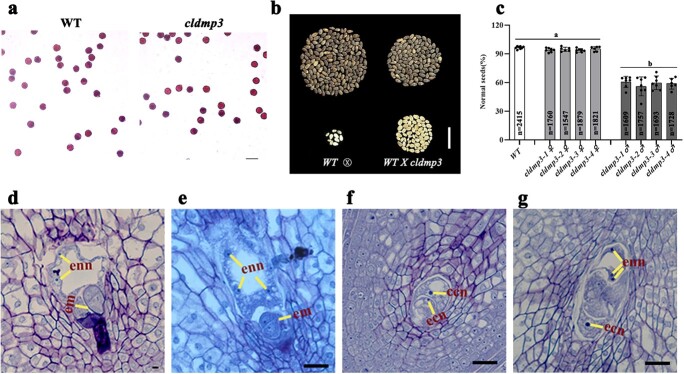
Phenotypic analysis of the *cldmp3* mutants. **a.** Pollen viability comparison of wild-type and *cldmp3* mutant stained by Alexander staining. **b.** Watermelon seeds from selfing WT and a WT x *cldmp3* cross. Undeveloped seeds (white) are shown at the bottom of the image. **c.** Percentage of normal seeds when *cldmp3* mutants were used as female (♀) and male (♂) parents, respectively. Data are the means ± SD. *P* < 0.0001 (two-sided Student’s t-test). n represents the total number of seeds. **d-g.** Semi-thin paraffin sections of WT (d) and the cross progeny (WT x *cldmp3–2*) ovules (e-g) three days after pollination. Ecn, egg cell nuclei; ccn, central cell nuclei; em, embryo; enn, endosperm nuclei. Scale bars: 100 μm (a), 3 cm (b), 20 μm (d-g).

*cldmp3* pollen grains were fully viable when stained by Alexander solution, which ruled out the possibility of defective male gametophyte development (Fig. 4a). To further identify potential defects in male and female gametes of the mutants, reciprocal crosses were carried out between *cldmp3* and wild type (WT) plants. When the *cldmp3* mutants were used as male parents, there was a significant reduction in the number of normal seeds produced compared to selfed WT plants (Fig. 4b and c). However, the seed number was not affected when the mutants were pollinated with WT pollen (Fig. 4c), indicating that the mutation in *ClDMP* gene caused partial male sterility. The above results implied that the reduced seed-set while crossing to wild type could be caused by defective double fertilization. Since all mutants showed similar defects, the homozygous *cldmp3–2* mutant was chosen for further analysis.

To better understand the developmental process of double fertilization occurred in the mutants, semi-thin paraffin sectioning was carried out on WT ovules pollinated by the *cldmp3–2* mutant pollen grains. Three days after pollination (DAP), some ovules developed normal embryos and endosperm, which were indistinguishable from the wild type (Fig. 4d and e). However, some embryo sacs contained undeveloped egg cells and central cells, representing a complete failure of fertilization of both gametes (Fig. 4f). Notably, some embryo sacs contained only endosperm without detectable developing embryos at 3DAP (Fig. 4g), indicating single fertilization of the central cell by the sperm cell. Consistent with previous studies, our results showed that absence of the ClDMP3 protein resulted in single fertilization events of central cells in watermelon. It is therefore possible that a small portion of unfertilized egg cells could sense the abnormal sperm-egg cell interaction, and initiate parthenogenic embryogenesis to develop into maternal haploid embryos.

### Haploid screening process in watermelon

**Figure 5:**
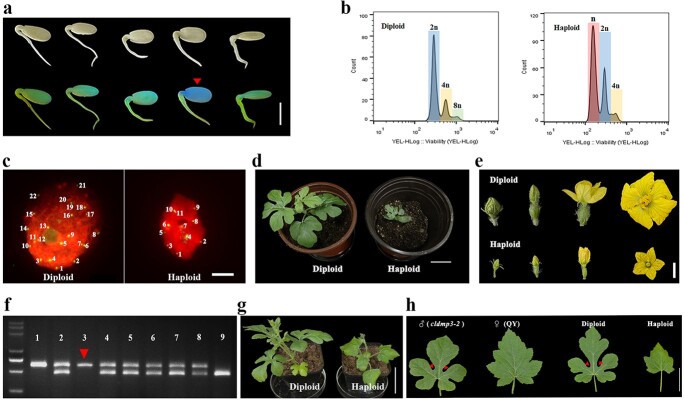
Identification of haploids induced by the *cldmp3* inducer line. **a.** Germinated seeds under bright field and green fluorescence. The red arrow indicates the potential haploid seed with no fluorescence. **b.** Flow cytometry verification of diploid and haploid plants. **c.** Immuno-staining of diploid and haploid nuclei using a watermelon centromere-specific CenH3 antibody. DNA was counterstained with DAPI (in red). Centromere signals (in green) indicate the chromosome number (diploid 2n = 22; haploid n = 11). **d.** Diploid and haploid plants in the F1 progeny of the “LLW” x *cldmp3–2* crosses. **e.** Flower buds of diploid and haploid plants at different stages. **f.** Genotyping of diploid and haploid plants using the CAPS maker on Chromosome 3. Lane 1 is the female parent and lanes 2–8 are the progeny plants of “97 103” x *cldmp3–2*. Lane 9 is the male parent. The red triangle indicates the haploid. **g.** Diploid and haploid plants identified from F1 progeny of “QY” x *cldmp3–2* crosses. **h.** Leaf shape comparison of diploid and haploid plants identified from the F1 progeny of *cldmp3–2* x “QY” crosses. The red triangles indicate the lobed edge of the leaves. Scale bars: 1 cm (a and e), 5 μm (c), 5 cm (d and g), 3 cm (h).

To confirm the haploid induction ability of *cldmp* mutants, the *cldmp3–2* mutant was used as a pollen inducer that was crossed with the hybrid “linglongwang” (LLW). Since the GFP expression cassette was integrated into the *cldmp3* mutant genome along with the T-DNA of CRISPR/Cas9 vector, we utilized it as a marker for hybrid seeds. Seeds with no fluorescence were therefore considered to be potential haploids, due to the fact that haploids induced by the *cldmp3* mutant should be maternal and not inherit GFP from the male side (Fig. 5a). Flow cytometry was then utilized to check the ploidy level of the plants. Compared to the 2C, 4C and 8C peaks in the WT plants, haploid plants contained 1C, 2C and 4C peaks (Fig. 5b), which was indicative of a reduction of DNA ploidy. Immunofluorescence staining experiments were carried out with a centromere-specific CenH3 protein antibody to further confirm the chromosome number in haploids. This analysis detected 22 signals in the diploid WT watermelon plants (2n = 22) but only 11 fluorescence signals in the haploid plants (n = 11) (Fig. 5c), which further confirmed that chromosome number of the haploid was half that of the WT. Phenotypic analysis indicated that the haploids grew more slowly than the diploids, and had smaller leaves and floral organs (Fig. 5d and e). Additionally, haploid plants were sterile due to the uneven segregation of the chromosomes during meiosis. A total of four haploid plants were identified from 356 F1 hybrid seeds, representing a 1.12% haploid induction rate (HIR).

To further test the haploid induction ability of *cldmp3* in different genotypes, *cldmp3–2* was used as the male parent in a cross with “97 103” and non-lobed leaf watermelon material “QY” [38]. Cleaved amplified polymorphic sequence (CAPS) markers were developed on all 11 chromosomes of “97 103” (Table S1). During haploid screening of the F1 generation, the hybrids displayed two bands, whereas the haploids contained only one maternal band (Fig. 5f). Additionally, analysis of all 11 chromosomal markers confirmed the haploids. The non-lobed leaf trait is known to be controlled by a single recessive gene, and *cldmp3–2* was created in the inbred “TC”, which has lobed leaves. Therefore, in the F1 progeny of “QY” x *cldmp3–2* crosses, the leaves of maternal haploids were round and non-lobed, which were visually distinguishable from the hybrids with lobed leaf edges at the seedling stage (Fig. 5g and h). The above visual recessive marker greatly simplified the haploid screening process. Analysis of this phenotype revealed that *cldmp3–2* induced haploids at rates of 0.60% and 0.51% in “97 103” and “QY”, respectively, demonstrating a broad range of *DMP*-mediated haploid induction across different watermelon genotypes (Table 1).

**Table 1:** Haploid induction rate (HIR) of the *cldmp3–2* inducer line

**Female parent**	**Total seeds**	**Haploids**	**Haploid Induction Rate (HIR)**
**LLW**	**356**	**4**	**1.12%**
**97 103**	**498**	**3**	**0.60%**
**QY**	**592**	**3**	**0.51%**

## Discussion

Haploid induction systems have the potential to greatly accelerate plant breeding processes through the generation of homozygous DH lines in one generation. Since irradiated pollen grains can induce parthenogenic haploids in cucurbits crops at low frequencies, we hypothesized that the irradiated pollen grains cause preferential fertilization of the central cell over the egg cell, which leads to haploid formation. In this study, we have shown that mutation of the *ClDMP3* gene, which encodes a DUF679 protein that is localized to the sperm membrane, can induce watermelon haploids at rates of 0.51 to 1.12%. Recently, Xu’s group also developed a haploid induction system in watermelon by utilizing *cldmp* mutants, with a similar haploid induction rate (1.08%) [39]. Both Xu’s and our results clearly show that the *DMP*-mediated haploid induction technique is feasible in watermelon, with significant implications for the development of new breeding strategies in watermelon that could lead to the production of valuable watermelon DH lines.

### Further optimization of the watermelon transformation system

In order to successfully generate haploid inducer lines in watermelon at large scales, the transformation system, which facilitates the generation of mutant plants, needs further optimizations. Since the establishment of transformation systems in cucurbit crops, significant effort has been put into optimizing these processes [40–42]. Plant growth regulator genes, including *BBM*, *GRF*, *WUS* and *WOX5*, have been proven to boost transformation efficiencies in many plant speces [43–47], and the chimeric *GRF4-GIF1* gene has been shown to increase transformation efficiency up to nine fold in a genotype-independent manner in watermelon [48]. Although constitutive overexpression of *GRF4-GIF1* by the 35S promoter increases regeneration rates, it also results in malformed tissue during transformation (data not shown). It is possible that switching to a less active promoter could fix this issue, but additional research is needed to identify candidates. Callus or tissue-culture specific promoters are other possible strategies to avoid pleiotropic effects in later developmental stages caused by the overexpression of the growth regulator genes [49]. However, no callus-specific promoters have been reported in dicot plants. In addition to low efficiency, watermelon transformation also has a long cycle time, requiring an average of four to five months to obtain transgenic plants. Furthermore, rooting is one of the limiting steps of this process, taking up to 1.5 months. The development of more efficient rooting protocols or grafting positive transgenic shoots onto rooted stock plants have the potential to dramatically accelerate this process.

### Precision genome editing tools are needed in watermelon

Since the establishment of the first CRISPR/Cas9 system in watermelon, it has been widely applied in breeding and gene function characterization [40, 50, 51]. More recently, an herbicide-resistant watermelon variety was created by employing the CRISPR/Cas9 system to edit the *ALS* gene [41]. With the rapid development of genomics and pan-genomics, more essential SNPs for agronomic traits have been identified and more efficient base editors are therefore needed to genetically engineer crops. For haploid induction, amino acid substitutions in the CenH3 protein have proven to be an effective strategy to induce haploids in model plants [9–11]. Efficient base editors and the PRIME editing system have the potential to significantly accelerate the development of inducer lines in watermelon [52].

### The haploid screening process needs to be simplified in watermelon

Currently, the haploid screening process is dependent on fluorescent protein markers to identify haploids during the seed or seedling stage. Identifying the haploid at the seed or seedling stage could greatly simplify the process and result in time and labor savings. However, the 35S-driven GFP construct cannot stably propagate across generations in watermelon (data not shown), likely due to silencing of the GFP-cassette. A new marker system is therefore needed if extensive haploid screening is to be carried out. Fluorescent protein markers driven by seed-specific promoters represent one possible solution [53]. Another strategy is to use recessive phenotypic or molecular markers to simplify the process, such as the non-lobed leaf marker and CAPS markers used in this work. However, the ultimate goal of this process is to induce haploids in the hybrid backgrounds, which makes the above strategies less useful. Chromosome number confirmation is also necessary for haploid identification because aneuploids can be generated during some induction processes, such as the CenH3-based approach. Currently, there are two major methods to determine chromosome number, including chromosome counting by cytological staining [39] and immuno-staining. Immuno-staining is a two-day process and does not require pretreatment of the samples, which makes it possible to simultaneously handle a large number of samples.

In this work, we present a detailed method of establishing the haploid induction system in watermelon. Additional work is still required to increase the efficiency of this system and make it more applicable to commercial DH lines used in watermelon breeding.

## Materials and methods

### Plant material

The wild watermelon variety “TC” used in this experiment was collected in Tongchuan (Shaanxi province). “97 103” was provided by Dr. Yong Xu’s lab (Beijing Academy of Agricultural and Forestry Sciences) and inbred for at least four generations. “LLW” is a hybrid variety and “QY” is a self-crossing offspring identified from the selfing progeny of the commercial watermelon hybrid “Lingxiu”. All the above materials were planted in experimental fields of Northwest A&F University, Yangling.

### Phylogenetic analysis

The DMP protein sequences from *Nicotiana tabacum*, *Solanum lycopersicum* (https://www.ncbi.nlm.nih.gov/), *C. lanatus* (http://cucurbitgenomics.org/), *Arabidopsis thaliana* (https://www.arabidopsis.org/), *B. napus* (http://www.brassicadb.cn/), *Glycine max* (https://www.soybase.org/), *Z. mays* (https://www.maizegdb.org/) and *Oryza sativa* (https://rapdb.dna.affrc.go.jp/) were used in the construction of phylogenetic trees. Protein IDs are listed in Table S2. Multiple sequence alignment of the DMP proteins was conducted using ClustalW with default parameters. The misaligned proteins portions before and after the central protein sequence were removed manually. A neighbor-joining tree was constructed with 1000 bootstrap replicates using MEGA 11 software [54].

### Subcellular localization of ClDMP3

The cDNA sequence of *ClDMP3* was amplified using gene-specific primers (Table S3) and ligated into the pGREEN vector to fuse with a green fluorescent protein (GFP) gene. The fusion protein ClDMP3-GFP was driven by a 35S promoter. AtCBL-RFP was also driven by a 35S promoter and was used as the membrane maker [55]. The two constructs were transiently co-transformed into tobacco (*Nicotiana benthamiana*) leaves through infiltration with *Agrobacterium* strain GV3101 (containing helper plasmid pSoup). After 48 hours, a confocal laser scanning microscope (Leica TCS-SP8 SR, Germany) was used to observe fluorescence.

### Whole-mount RNA *in situ* hybridization of pollen grains

Whole-mount RNA *in situ* hybridization was performed as described previously, with minor modifications [56]:

1. The full-length cDNA of *ClDMP3* was used to prepare the RNA probes.
2. A DIG RNA Labeling Kit (Roche, Rotkreuz, Switzerland) was used to label the sense and antisense probes of *ClDMP3*, according to the manufacturer’s instructions.
3. Fixation: Pollen grains were collected or germinated in an Eppendorf tube containing 4% paraformaldehyde in PBS (pH 7.4) at room temperature for 2–3 hours.
4. Samples were dehydrated with rinses of 3:7, 1:1 and 7:3 ME: PP (ME = 90% methanol-10% 0.5 M EGTA; PP = 4% formaldehyde in PBS containing 130 mM NaCl-10 mM NaH_2_PO_4_, pH 7.4) and 2 x 100% ethanol. Each rinse was carried out for 30 minutes, followed by 30 minutes of 100% xylene rinsing.
5. Rehydration was carried out with an 85%, 70%, 50%, 30% and 10% water series, 10 minutes each.
6. Proteinase treatment: The sample was incubated in Proteinase K buffer: 1 μg/mL proteinase K, 100 mM Tris–HCl (pH 7.5), 50 mM EDTA at 37°C for 35 minutes.
7. The sample was washed with PBS + 2 mg/mL Glycine for 2 x 5 minutes.
8. Refixation: The sample was incubated in 4% formaldehyde in PBS for 20 minutes and washed for 2 x 5 minutes in PBS.
9. Prehybridization: Samples were incubated in prehybridization buffer: 6 x SSC, 0.1% SDS, 50% formamide, 100 μg/mL tRNA at 42°C for 3 hours.
10. Hybridization: DIG-labeled RNA probes were added to a final concentration of 500 ng/mL and incubated at 42°C overnight.
11. Samples were washed in 2 x SSC, 0.1% SDS at 42°C for 10 minutes.
12. RNase treatment: Samples were incubated in 150 μL 10 mM Tris–HCl (pH 8.0), 0.5 M NaCl, 1 mM EDTA and 40 μg/mL RNase A at 37°C for 30 minutes.
13. Samples were washed with 1 x SSC, 0.1% SDS and 0.5 x SSC-0.1% SDS for 3 x 10 minutes at 42°C.
14. Blocking of the samples was carried out with 1% blocking reagent and 0.3% Triton X-100 for 1 hour at room temperature.
15. The sample was incubated in PBS-3% BSA with anti-DIG-AP conjugate for 1–2 hours and washed 3 x 20 minutes in PBS.
16. For detection, the sample was incubated with BCIP/NBT solution until a signal appeared.

### Vector construction

The pBSE402 gene editing vector was used to create the *cldmp3* mutant. A single guide RNA was designed by CRISPR-P v2.0 (http://crispr.hzau.edu.cn/cgi-bin/CRISPR2/CRISPR) and cloned into the pBSE402 vector [48]. The colony-PCR-positive clones were further confirmed by Sanger sequencing. The plasmid was isolated and transformed into the *Agrobacterium* strain EHA105 for watermelon transformation.

### Watermelon transformation

1. Healthy watermelon seeds were soaked in distilled water at 50–55°C for 30 minutes, then deshelled. The deshelled seeds were placed on germination medium (BM, breeding medium; agar 4.43 g/L) and incubated in a petri dish at 28°C in the dark for 1–2 days.
2. After the seeds were evenly germinated, two cotyledons were cut into eight pieces to serve as explants. Simultaneously, a single EHA105 agrobacteria colony was grown to OD600 = 0.8–1.0. Excised cotyledons were rinsed in diluted bacterial cultures for 15 minutes (OD600 = 0.08–0.1), then transferred onto a filter paper to drain out the extra liquid. The explants were then transferred and cultivated in medium (MS519 4.43 g/L, sucrose 30 g/L, agar 3 g/L, 6-BA 1.5 mg/L) in the dark at 28°C for three days before being moved to normal growth conditions with 18 hours of daylight and 6 hours of darkness.
3. Explants were transferred to shoot generation medium (MS519 4.43 g/L, sucrose 30 g/L, agar 3 g/L, 6-BA 1.5 mg/L, 200 mg/L Timentin) and placed at 28°C for 10–12 days. Fluorescence was visualized with a stereoscopic fluorescence microscope (MZ10F, Leica, Germany), and positive shoots that contained GFP fluorescence were labeled. When the positive shoots had grown to 1–2 cm, they were transferred to new shoot generation medium.
4. Healthy positive shoots were transferred onto rooting medium (MS519 4.43 g/L, sucrose 30 g/L, agar 3 g/L, 200 mg/L Timentin). When 4–5 fresh leaves had formed on the shoots, the transgenic plants were transferred to the soil to continue growth.
5. DNA was extracted from positive shoots. After amplifying the target region with gene-specific primers, Sanger sequencing and Hi-TOM deep sequencing were used to confirm the editing types and ratios.

### Alexander staining

Fresh flowers were collected to spread pollen evenly onto glass slides with approximately 20 μL Alexander’s staining solution, and incubated for 2–3 hours after the application of cover slips [57]. An Axiocam light microscope (Zeiss) was used to observe the pollen viability.

### Semi-thin paraffin sectioning

Semi-thin paraffin sectioning was carried out using 7100 and 3040 embedding kits purchased from the Kulzer Technovit company, with the following steps:

1. Ovules were collected three days after pollination and fixed in FAA solution for infiltration at 4°C for 24 hours.
2. Samples were dehydrated by washing them with a 20%, 40%, 60%, 80% and 100% ethanol series for 30 minutes per wash.
3. 100% ethanol: 7100 base liquid (1:1) mixed solution was used to pre-infiltrate the samples for 2–3 days.
4. One gram of hardener I was diluted into 100 mL of 7100 base solution, and the infiltrate solution was added to the sample after pouring out the pre-infiltrated solution from the previous step.
5. A total of 1 mL of hardener II was diluted into 15 mL of infiltrate solution, and the solution was added to each sample after the tissues were placed into the mold and covered with parafilm to exclude air for overnight incubation.
6. The parafilm was removed when the samples had settled. A glue solution (1:2 volume of 3040 liquid: 3040 powder) was used to attach the embedded tissues and the sectioning blocks.
7. The ovules were sectioned to a thickness of 3 μm using a glass knife. After stretching the samples out on water, they were placed on a 60°C hotplate for two hours. Then, 1% TBO was added to stain the samples for 1–2 minutes.

### Flow cytometric analysis

Fresh true leaves were collected from watermelon seedlings, then a razor blade was used to chop the leaves (1 cm x 1 cm) in nuclei isolation buffer. The suspension solution was filtered through a 35 μm cell filter, and the flow-through nuclei were collected. The nuclei solution was treated with 50 μg/mL RNaseA, then stained by adding 50 μg/mL PI (Propidium Iodide) for 30 minutes [58]. Flow cytometry analysis was performed using a Muse Cell Analyzer (Luminex), according to the manufacturer’s direction.

### Marker development

The whole genome sequence of “TC” was combined with data from the Cucurbit Genomics Database (CuGenDB, http://cucurbitgenomics.org/) to identify SNP (single nucleotide polymorphism) loci on each chromosome. CAPS markers were then developed for reliable SNP loci confirmed by PCR and sequencing. Primers were designed using Geneious software (http://www.geneious.com). The primers of the markers are shown in Table S1.

### Immuno-staining

1. CenH3 protein antibody was raised against the N terminus protein sequence, which was manufactured by Genscript (Hangzhou).
2. A total of 10 mg of leaf tissue was fixed in 10 mL ice-cold 4% paraformaldehyde in Tris-buffer for 1 x 5 minutes under vacuum. Fixation was carried out for 20 minutes in ice-cold fixative.
3. Two 10-minute rinses with ice-cold Tris buffer were carried out.
4. The tissue was sliced in 1000 μL ice-cold LB01 buffer in a pre-cooled Petri-dish with a razor blade.
5. A 15 μL suspension was added to microscope slides with a pipet tip in circular movements. Slides were then left to dry at room temperature overnight.
6. The slide glass was washed twice with 1 x PBS for five minutes at room temperature.
7. 60 μL of 1^st^ antibody solution (2% BSA in 1 x PBS with 0.1% Tritonex-100:1^st^ antibody = 200:1) was dropped onto the slide, which was then covered with parafilm. The slide was kept at high humidity overnight at 4°C.
8. The slide glass was washed two times with 1 x PBS for five minutes at room temperature.
9. 60 μL of 2^nd^ antibody solution (2% BSA in 1 x PBS with 0.1% Tritonex-100:2^st^ antibody = 200:1) was dropped onto the slide, then left at high humidity at 37°C for one hour.
10. The slide glass was then washed two times with 1 x PBS for five minutes at room temperature.
11. The sample was dehydrated with two-minute sequential washes of 70%, 90% and 100% ethanol at room temperature. The slide was then left to dry at room temperature.
12. Samples were stained with DAPI.

## Supplementary Material

Web_Material_uhad081

## Data Availability

All data generated in this study are presented in the paper. Additional data related to this paper may be requested from the authors.
