# Supplementary material for: Creation of a watermelon haploid inducer line via ClDMP3-mediated single fertilization of the central cell
Source: Hortic Res. 2023 Apr 19;10(6):uhad081. doi: 10.1093/hr/uhad081 (PMC10261877; doi:10.1093/hr/uhad081)
Supplement: Web_Material_uhad081 [file web_material_uhad081.docx]

**Fig. S1** Expression analysis of *ClDMP3* in different tissues

| Makers | Product size/bp | Restriction enzyme | Primer Sequence (F) | Primer Sequence (R) |
| --- | --- | --- | --- | --- |
| Chr01 | 543 | SacⅠ | CCTGCTGGGGTTAGCTCTTTTAT | CATGCACACTGTTCTGAGCCAA |
| Chr02 | 594 | Hindlll | CCTATCAAGTCGGCTGCAACAT | CCAACTCATTGGTTCTATACCACG |
| Chr03 | 608 | PstⅠ | CATGCACCCCACAAACAAACT | GCTAAGGGAATTCTGTACCTGCAC |
| Chr04 | 564 | HindⅢ | GGAGGTGGTCTCAAACACTTTTAG | CATCTGCTCAACTGGTGTCCTTCT |
| Chr05 | 695 | EcoRⅠ | GGGGAAAACTAACCTAAAACG | GCAGCAAGTTAGCCATTGGCAT |
| Chr06 | 763 | XhoⅠ | GTCTCGGCTGCAAGTGTCAATCT | GATCATAGCATTCGGTATCAG |
| Chr07 | 719 | HindIII | CCGTTGGGCTTGCACAATGAT | ATATTCCACCCATTCTCTAGC |
| Chr08 | 467 | XhoⅠ | GGTGTTGCTCAGTCCTACAAAC | GCAGCAGTTAATAGCCAACTGATC |
| Chr09 | 667 | XhoⅠ | TGGAGAATTGGGGAAAGAAGCG | TGGAGTAGCTGAAGTTTTGG |
| Chr10 | 723 | PstⅠ | CCACCCTCAAAGAATTCTTCC | AGCCCTTACCTTTGGCAAACC |
| Chr11 | 486 | EcoRⅠ | GTCAATGCAAAGGTAGAATC | GACCGTTCTTGTATGTAATGTG |

**Table S1** Primer information for the CAPS markers

| **Table S2** DMP family proteins used in this study | | |
| --- | --- | --- |
| DUF679 membrane proteins in other species | Protein IDs | Name |
| *Arabidopsis thaliana* | AT3G21520.1 | DMP1 |
| *Arabidopsis thaliana* | AT3G21550.1 | DMP2 |
| *Arabidopsis thaliana* | AT4G24310.1 | DMP3 |
| *Arabidopsis thaliana* | AT3G21550.1 | DMP4 |
| *Arabidopsis thaliana* | AT3G02430.1 | DMP5 |
| *Arabidopsis thaliana* | AT5G46090.1 | DMP6 |
| *Arabidopsis thaliana* | AT4G28485.2 | DMP7 |
| *Arabidopsis thaliana* | AT1G09157.1 | DMP8 |
| *Arabidopsis thaliana* | AT5G39650.1 | DMP9 |
| *Arabidopsis thaliana* | AT5G27370.1 | DMP10 |
| *Solanum lycopersicum* | XP_004233838.1 |  |
| *Solanum lycopersicum* | XP_004248460.2 |  |
| *Solanum lycopersicum* | XP_004230455.1 |  |
| *Solanum lycopersicum* | XP_004248908.1 |  |
| *Solanum lycopersicum* | XP_004239396.1 |  |
| *Solanum lycopersicum* | XP_004229478.1 |  |
| *Zea mays* | Zm00001d034995 |  |
| *Zea mays* | Zm00001d016492 |  |
| *Zea mays* | Zm00001eb383510 |  |
| *Zea mays* | Zm00001d044777 |  |
| *Zea mays* | Zm00001d044822 | ZmDMP |
| *Zea mays* | Zm00001d044435 |  |
| *Zea mays* | Zm00001d008235 |  |
| *Zea mays* | Zm00001eb343640 |  |
| *Oryza sativa* | LOC_Os01g27070.1 |  |
| *Oryza sativa* | LOC_Os01g27100.1 |  |
| *Oryza sativa* | LOC_Os01g27120.1 |  |
| *Oryza sativa* | LOC_Os01g28080.1 |  |
| *Oryza sativa* | LOC_Os01g28089.1 |  |
| *Oryza sativa* | LOC_Os01g29240.1 |  |
| *Oryza sativa* | LOC_Os01g29280.1 |  |
| *Oryza sativa* | LOC_Os01g29330.1 |  |
| *Oryza sativa* | LOC_Os01g65992.1 |  |
| *Oryza sativa* | LOC_Os02g27800.1 |  |
| *Oryza sativa* | LOC_Os03g25440.1 |  |
| *Oryza sativa* | LOC_Os05g48840.1 |  |
| *Oryza sativa* | LOC_Os06g24490.1 |  |
| *Oryza sativa* | LOC_Os07g22510.1 |  |
| *Oryza sativa* | LOC_Os07g45080.1 |  |
| *Oryza sativa* | LOC_Os08g01530.1 |  |
| *Oryza sativa* | LOC_Os12g22270.1 |  |
| *Glycine max* | Glyma.02g075800.1 |  |
| *Glycine max* | Glyma.06g288800.1 |  |
| *Glycine max* | Glyma.07g201500.1 |  |
| *Glycine max* | Glyma.07g201500.2 |  |
| *Glycine max* | Glyma.07g253500.1 |  |
| *Glycine max* | Glyma.07g253600.1 |  |
| *Glycine max* | Glyma.09g237500.1 |  |
| *Glycine max* | Glyma.13g175000.1 |  |
| *Glycine max* | Glyma.13g212400.1 |  |
| *Glycine max* | Glyma.13g235100.1 |  |
| *Glycine max* | Glyma.16g157800.1 |  |
| *Glycine max* | Glyma.17g020800.1 |  |
| *Glycine max* | Glyma.17g020900.1 |  |
| *Glycine max* | Glyma.18g097400.1 |  |
| *Glycine max* | Glyma.18g098300.1 | GmDMP9 |
| *Nicotiana tabacum* | XP_016487246.1 | NtDMP9-like a |
| *Nicotiana tabacum* | XP_016436254.1 | NtDMP9-like b |
| *Nicotiana tabacum* | XP_016459518.1 | NtDMP9-like c |
| *Nicotiana tabacum* | XP_016497787.1 |  |
| *Nicotiana tabacum* | XP_016496141.1 |  |
| *Nicotiana tabacum* | XP_016434048.1 |  |
| *Nicotiana tabacum* | XP_016514025.1 |  |
| *Nicotiana tabacum* | XP_016474928.1 |  |
| *Nicotiana tabacum* | XP_016456784.1 |  |
| *Nicotiana tabacum* | XP_016501846.1 |  |
| *Nicotiana tabacum* | XP_016441817.1 |  |
| *Citrullus lanatus* | Cla97C06G121370 |  |
| *Citrullus lanatus* | Cla97C01G023140 |  |
| *Citrullus lanatus* | Cla97C02G036040 |  |
| *Citrullus lanatus* | Cla97C05G087060 |  |
| *Citrullus lanatus* | Cla97C05G087050 |  |
| *Citrullus lanatus* | Cla97C05G087040 |  |
| *Brassica napus* | BnaA03g55920D |  |
| *Brassica napus* | BnaC03g03890D |  |
| *Brassica napus* | BnaA04g09480D |  |
| *Brassica napus* | BnaC04g31700D |  |
|  |  |  |

**Table S3** Primers used for gene expression analysis and vector construction

| Experiment | Primer name | Primer Sequence (5'-3') |
| --- | --- | --- |
| For RT-PCR | sRTDMP1370-F | ATGGATGAACACACACTAACCACCG |
|  | sRTDMP1370-R | TCAATTGGCCATGCAACCAATGCC |
|  | sRTDMP3140-F | ATGGAGATCAAAGCTGCCGACGAAGA |
|  | sRTDMP3140-R | CTAATTAGCAGAGATTGGGAAGCC |
|  | sRTDMP6040-F | ATGGAAGGGGAAATCGAGAGCCAGAT |
|  | sRTDMP6040-R | CTACTGACGAGAGAGGGGGAATCCAA |
|  | sRTDMP7060-F | TCTGCGGTCTCTCCTGTTTTCTCTCC |
|  | sRTDMP7060-R | CACCACGCTAGACACCGCTCCAACG |
|  | sRTDMP7050-F | ATGGCCACCTCCCAGTTTAACCAAAA |
|  | sRTDMP7050-R | TCAAAGCCCTATGCCATGGCGAGTGT |
|  | sRTDMP7040-F | ATGGCCACCTCCGGGAAGCAA |
|  | sRTDMP7040-R | TTAGCTTAATATAGCCTTTTGAACTC |
|  | sRTACTIN-F | ATGGCTGATGCTGAGGATATCCAG |
|  | sRTACTIN-R | CATACCCACCATGACACCAGTGTGT |
|  | qRTDMP1370-F | CACGCCGTTATGTCTGTGATG |
|  | qRTDMP1370-R | ATGCCATATCTAGTATTGGGAAACAC |
|  | qRTACTIN-F | CCTACAACTCAATTATGAAGTGTG |
|  | qRTACTIN-R | GAAATCCACATCTGCTGGAAGGTG |
| For Subcellular location | DMP-PGREEN-F | TCGATAAGCTTGATATCATGGATGAACACACACTAACCACCG |
|  | DMP-PGREEN-R | TCCTTTACTCATACTAGTATTGGCCATGCAACCAATGCC |
| For RNA in situ hybridization | DMP-insitu-F | TGTAATACGACTCACTATAGGGATGGATGAACACACACTAACC |
|  | DMP-insitu-R | GATTTAGGTGACACTATAGAATGCTGAAGTTGACTAGCATCGAGGTT |
| For gene editing | DMP-gRNA-F | ATTGCTAGTCAACTTCCTTCCAAC |
|  | DMP-gRNA-R | AAACGTTGGAAGGAAGTTGACTAG |
